# Proteomic comparison reveals the contribution of chloroplast to salt tolerance of a wheat introgression line

**DOI:** 10.1038/srep32384

**Published:** 2016-08-26

**Authors:** Wenjing Xu, Hongjun Lv, Mingming Zhao, Yongchao Li, Yueying Qi, Zhenying Peng, Guangmin Xia, Mengcheng Wang

**Affiliations:** 1 The Key Laboratory of Plant Cell Engineering and Germplasm Innovation, Ministry of Education, School of Life Science, Shandong University, 27 Shandanan Road, Jinan, Shandong 250100, China; 2 Bio-Tech Research Center, Shandong Academy of Agricultural Science, Shandong Provincial Key Laboratory of Genetic Improvement, Ecology and Physiology of Crop, Jinan, 250100, China

## Abstract

We previously bred a salt tolerant wheat cv. SR3 with bread wheat cv. JN177 as the parent via asymmetric somatic hybridization, and found that the tolerance is partially attributed to the superior photosynthesis capacity. Here, we compared the proteomes of two cultivars to unravel the basis of superior photosynthesis capacity. In the maps of two dimensional difference gel electrophoresis (2D-DIGE), there were 26 differentially expressed proteins (DEPs), including 18 cultivar-based and 8 stress-responsive ones. 21 of 26 DEPs were identified and classified into four categories, including photosynthesis, photosynthesis system stability, linolenic acid metabolism, and protein synthesis in chloroplast. The chloroplast localization of some DEPs confirmed that the identified DEPs function in the chloroplast. The overexpression of a DEP enhanced salt tolerance in *Arabidopsis thaliana*. In line with these data, it is concluded that the contribution of chloroplast to high salinity tolerance of wheat cv. SR3 appears to include higher photosynthesis efficiency by promoting system protection and ROS clearance, stronger production of phytohormone JA by enhancing metabolism activity, and modulating the *in chloroplast* synthesis of proteins.

Soil salinity is one of the most common abiotic stresses constraining crop growth and productivity. Chloroplast is not only a factory for energy assimilation but also the site for synthesis of abscisic acid (ABA), jasmonic acid (JA) and other phytohormones and important metabolites. ABA and JA have been widely reported to play crucial roles in the response to abiotic stresses. Moreover, chloroplast possesses its own genome, and can emit retrograde signals to coordinate the expression of nuclear genes with the metabolic and developmental state of the cell^1^,^2^,^3^. Especially, in chloroplast, ROS are produced as an unavoidable side effect of the photosynthetic light reactions^4^,^5^. ROS generation in chloroplasts is enhanced by environmental stress influencing the photosynthetic efficiency^6^,^7^, which in turn changes the redox state of the plastid^8^. The redox state is also instrumental in regulating the chloroplast metabolic activities but also plastid and nuclear gene expression^1^,^9^,^10^. Based on these, chloroplast is closely associated with salt response in plants, and serves as an excellent model for better understanding redox system and the mechanism of the response. Thus, it is necessary to apply effort to gain a better understanding of the adaptive mechanisms of chloroplast used by plants to combat abiotic stress, with aim to improve tolerance of crops.

Wheat is one of the major crops in the world, and has been subjected to intensive breeding and selection. However, wheat is a glycophyte, and lacks tolerance to high salinity. We previously bred the bread wheat cultivar SR3 (or simply SR3) with high salt tolerance, which was selected from a wheat introgression line via asymmetric somatic hybridization with the bread wheat cv. JN177 (or simply JN177) as the recipient and its wild relative tall wheatgrass (one of the most salinity tolerant monocotyledonous species^11^) as the donor^12^. SR3 genome was introgressed with six chromatin fragments of tall wheatgrass^13^, in which high frequency of genetic and epigenetic variation has taken place^14^,^15^. Thus SR3 is a special wheat mutant and can be used to mine some associated genes closely responding to abiotic stress. For instance, *TaSRO1*, the putative gene of SR3’s salt tolerance QTL, and *TaCHP* possess allelic variation between SR3 and JN177^16^,^17^, so genome-wide genetic and epigenetic variation contributes largely to the salt tolerance^18^.

Given that abiotic stress tolerance is a quantitative trait, high-throughput genetic screening platforms have delivered substantial insights into the landscape of response to the stresses. We previously performed proteomic and transcriptomic analysis, and found the salt tolerance of SR3 is partially attributed to the superior capacity of redox maintenance and carbon assimilation^19^,^20^,^21^, both of which are closely associated with chloroplast. Moreover, alike nuclear genome, the organellar genomes also generated genetic variation^22^, and some wheat introgression lines possess the chloroplast of donor species^23^,^24^,^25^, which may affect the chloroplast omics profiles and the nucleo-cytoplasmic interreaction. Thus, in this work, we conducted a proteomic comparison between SR3 and JN177, and decoded the basis of superior carbon assimilation efficiency of SR3 under the high salinity condition.

## Material and Methods

### Salinity and drought treatments

Wheat seedlings were grown in the half strength Hoagland's culture solution. The salinity treatment was applied to seedlings of SR3 and JN177 at the three-leaf stage by adding 200 mM NaCl to the half strength Hoagland's culture solution. Control plants remained in culture solution without any stress-inducing additive. After 24 h exposure, the leaves were harvested. All analyses were performed on three replicated plant samples.

### Physiological characterisation

The net photosynthesis rate of the second seedling leaf were assessed using a LI-6400XT Portable Photosynthesis System (LI-COR Biosciences), under 800 μmol·m^−2^·s^−1^ of light, and at 27 °C with a relative humidity of 40%. The leaves were subjected to dark-adaptation by placing in dark room overnight, and then were used to measure original fluorescence (*Fo*). The dark-adapted leaves were given a saturation pulse radiation (5000 μmol·m^−2^·s^−1^), and the maxium fluorescence (*Fm*) were measured. The potential maximum photochemical efficiency of PSII of chloroplasts (*Fv/Fm*) was calculated as (*Fm – F*_*0*_)/*Fm*. Leaf chlorophyll was extracted by acetone, and the contents of chlorophyll a and b were determined spectrophotometrically at 663 and 645 nm, respectively. Leaves of seedlings were sampled to measure ribulose bisphosphate carboxylase/oxygenase activity according to Lee *et al*.^26^ and phosphoribulokinase activity according to Hurwitz *et al*.^27^.

Quantification of the JA and ABA content was performed by the liquid chromatography-mass spectrometry (LC-MS) method. A 1 g aliquot of fresh leaves sampled from wheat seedlings at three leaf stage was snap-frozen, powdered and extracted in 1 mL methanol. The homogenate was held overnight at 4 °C, and then centrifuged with 15,000 *g* for 10 min, and finally the supernatant was evaporated. The residue was dissolved in 200 μL 0.1 M sodium phosphate (pH 7.8) and passed through a Sep-Pak C_18_ cartridge (Waters, USA), using 50% methanol as the eluant. After re-evaporation, the residue was dissolved in 50 μL 20% acetonitrile, and then a 5 μL aliquot was injected into a LC20AD-MS8030 LC-MS/MS system (Shimadzu, Japan) equipped with a BEH C_18_ (100 × 2.1 mm, 1.7 μm) column (Waters, USA) at 30 °C. The mobile phase was comprised of 55% acetonitrile and 45% water (v/v), and the flow rate was 0.30 mL min^−1^. Mass spectrometry was operated in the negative mode (ionization: electrospray; capillary voltage: 3.5 kV; collision energy: 15 eV; desolvation temperature: 250 °C; *m/z*: 209.1/59).

### Chloroplast isolation

The chloroplast was isolated using the leaves of wheat seedling at three-leaf stage. 10 g leaves were sampled and placed in 100 mL ice-bathed grinding buffer which was comprised of 2 ml 50 × HEPES-OH (2.5 M, pH8.0), 10 ml 10 × sorbitol (3.3 M), 2 ml 50 × EDTA-Na2 (100 mM, pH8.0), 2 ml 50 × ascorbic acid (400 mM), 0.0606 g L-Cysteine, 0.05 g Bovine serum albumin, and 88 ml H_2_O_2_, and then were homogenized 10 s for five times with the time interval of 10 s using a PRO PRO250 homogenizer (PRO scientific Inc, USA). The homogenates were filtered using two layers of Miracle membrane to remove dregs. The extracts were purified on Percoll gradients (85%/40%) by centrifugating with 47400 g at 4 °C for 15 min. The intact chloroplasts at the interface of two gradients were collected, washed by washing buffer [2 ml 50 × HEPES-OH (2.5 M, pH8.0), 10 ml 10 × sorbitol (3.3 M), 2 ml 50 × EDTA-Na_2_ (100 mM, pH8.0), H_2_O_2_ 86 ml], and enriched by centrifugating with 2000 g at 4 °C for 15 min.

### Organellar enzyme activity measurement

Leaves were homogenized in homogenization buffer [400 μL 50 × HEPES-OH (2.5 M, pH8.0), 50 ml 50 × EDTA-Na_2_ (100 mM, pH8.0), H_2_O_2_ 49.1 ml] at 4 °C. Isolated chloroplasts was suspended in the same homogenization buffer and sonicated at 4 °C for 1 min. The homogenates of both leaves and isolated chloroplasts were centrifugated (3000 rpm) at 4 °C for 15 min, and the supernatant was retained. The protein content was measured with the Bradford method^28^. The activities of catalase, alkaline phosphatase, and acid phosphatase were measured using the Kits (S0051, P0321, P0326) according to the manual (Beyotime, China). Alcohol dehydrogenase activity was measured according to Ellis and Setter^29^.

### Chloroplast protein extraction

Isolated chloroplasts were ground in liquid nitrogen, and the powders were suspended in 20 mL homogenization buffer (see above section) at 4 °C for 1 h. Then trichloroacetic acid was added to the final concentration as 15% (w/v), and stayed at 4 °C for 1 h. After centrifugation (40000 g) at 4 °C for 1 h, the supernatant was removed, and the pellet was washed with acetone containing 1 mM PMSF and 0.07% w/v β-mercaptoethanol twice. The pellet was vacuum dried at −40 °C using a FREEZONE 6 freeze dry system (LABCONCO, USA), and dissolved in 400 μl protein lysis solution containing 7 M urea, 2 M thiourea, 4% w/v CHAPS, 65 mM DTT, 1 mM PMSF and 0.5% v/v biolytes (Bio-Rad). Insoluble materials were removed by centrifugation, and the protein concentration of the sample quantified using the Bradford method^28^.

### Two dimensional gel electrophoresis and mass spectrometry analysis

Two dimensional gel electrophoresis was conducted according to Wang *et al*.^21^. The iso-electronic focus (IEF) was conducted using 17 cm pH4-7 linear pH gradient IPG strip (Bio-Rad). The protein solution containing 600 μg proteins was diluted with lysis solution to 375 mL, and evenly coated in a channel of the rehydration tray (Bio-Rad). The IPG strip was carefully placed on the protein solution to avoid producing bubbles, covered by mineral oil, and rehydrated at 20 °C overnight. The rehydrated IPG strip was washed quickly by ultrapure H_2_O, slightly removed surface water with humid filter paper, transferred into the IEF groove (Bio-Rad), and covered with mineral oil. IEF was conducted on a Bio-Rad PROTEAN IEF cell at 20 °C with the following procedure: 500 V for 1 h, 1000 V for 1 h, linear increase to 3000 V within 3 h, linear increase to 8000 v within 6 h, stay at 8000 V up to 100 kVh. After IEF, the strip was equilibrated by soaking in 2% DTT in equilibration buffer (6 M urea, 2% SDS, 0.05 M Tris-HCl pH 8.8, 20% glycerol) for 20 min followed by 2.5% iodoacetamide in equilibration buffer in dark at room temperature. The equilibrated IPG strip was placed on the top edge of 10% SDS-PAGE gel to avoid producing bubbles, and sealed by 1% low melting point agarose. The SDS-PAGE was done on the PROTEAN II cell (Bio-Rad) at constant current setting of 5 mA/gel for 2 h followed with 15 mA/gel until the bromophenol blue tracking dye arrived at the bottom edge of the gel. After SDS-PAGE, the proteins in the 2-DE gel were visualised by Coomassie brilliant blue R-250 staining^30^. Gel image was digitalized with a gel scanner (Powerlook 2100XL, UMAX), and analyzed with PDQuest™ software package (Bio-Rad). The analysis was based on total densities of gels with the parameter of percent volume, and a significant difference in expression of a spot (namely differentially expressed protein, DEP) was declared if the mean abundance varied more than two fold using *t*-test.

The mass spectometry analysis followed Wang *et al*.^21^. DEPs detected in gels were manually excised. After washing with Millipore pure water for three times, the gel pieces were destained with 50 mM ammonium bicarbonate (ABC)/50% acetonitrile (ACN) for several times until the blue staining was thoroughly disappeared, and then dehydrated with ACN. The gel pieces were reduced with DTT (10 mM in 50 mM ABC) for 60 min at 56 °C, and alkylated with iodoacetamide (55 mM in 50 mM ABC) for 45 min at room temperature in dark. Finally, the gel pieces were washed with 50 mM ABC for three times, dehydrated with ACN, and vacuum dried at −40 °C using a FREEZONE 6 freeze dry system (LABCONCO, USA). For in-gel digestion, the dry gel pieces were rehydrated in 5 μL trypsin solution (containing 20 ng/mL SIGMA proteomics grade porcine-modified trypsin in the buffer: 40 mM ABC solution and 9% ACN) for 1 h at 47 °C, covered with 15 μL trypsin buffer, and incubated at 37 °C for 16 h. The digested supernatant was transferred into a new tube. The peptides in gel pieces were successively extracted with 5% trifluoroacetic acid (TFA)/50% ACN on a sonication bath for 5 min twice and ACN for 10 min. The extract was combined with the digestion supernatant. The supernatant was vacuum dried at −40 °C, and the pellet was resuspended with 3 μL 0.5% TFA/30% ACN and desalted on a Ziptip C18 micro column (Millipore). Peptide aliquot (0.5 mL) was plotted onto metal plate and covered with 0.5 mL of matrix solution containing 10 mg/mL w/v α-cycano-4-hydroxycinnamic acid (CHCA) in 0.1% TFA/50% ACN. After air-dry, MALDI-TOF analysis was conducted using 4700 plus MALDI TOF-TOF™ analyzer (Applied Biosystems, USA). Peaklist-generating and peak-picking for MS data were conducted with GPS explorer (Applied Biosystems, 2006). Parameters and thresholds used for peak-picking were S/N threshold > 20, resolution > 10000 and means of calibrating each spectrum as external calibration. MS data were used to derive protein identity using the MASCOT search engine (http://www.matrixscience.com) applied to the NCBInr, MSDB and SwissProt databases. MS acceptance criterion is probability/E-value based scoring. The search parameters were one trypsin mis-cleavage permitted, MH^ + ^mass values and monoisotopic.

### Quantitative reverse transcription PCR (qRT-PCR)

The total RNA were isolated from leaves at three-leaf-stage seedlings under the control condition and treated by 200 mM NaCl for 24 h using the Trizol^®^ reagent (Invitrogen). The first cDNA strand was synthesized using an M-MLV reverse transcription system kit (Invitrogen) according to the manufacturer’s instructions. The cDNA was used as the template for 20 μL real-time PCR solution contained 10 μL 2 × SYBR Premix Ex Taq mix (Takara), 0.2 μM forward and 0.2 μM reverse primers, 1 μL of a 1:10 dilution of the cDNA first strand, and the cycling regime comprised a denaturation step of 95 °C for 2 min, followed by 45 cycles of 95 °C for 10 s, 60 °C for 20 s, 72 °C for 20 s. A melting curve analysis was performed over the range 80 °C to 95 °C at 0.5 °C intervals. Relative gene expression levels were detected using the 2^−ΔΔCT^ method^31^. A positive control was provided by a parallel analysis based on a fragment of the wheat *ACTIN* gene (AB181991), with three independent replicates per experiment.

### Isolation of DEP encoding genes

The sequence of each selected DEP was used as a tBLASTn query against the wheat EST database in NCBI. All matching ESTs were assembled to get a unigene using CAP3 software^32^, and a pair of specific primers designed from this assembly was used to amplify a full-length cDNA. The PCR procedure consisted of a 5 min denaturation at 95 °C, followed by 35 cycles of 94 °C for 30 s, 58 °C for 50 s and 72 °C for 60 s, with a final extension of 72 °C for 10 min. The amplicon was inserted into the pMD18-T vector (Takara), following the supplier's instructions, and submitted for sequencing.

### Subcellular localization

The full-length coding sequence, lacking its stop codon, was ligated into the 326-GFP vector. Either this recombined plasmid or the empty 326-GFP was transformed into *Arabidopsis thaliana* mesophyll protoplasts following the method described by Yoo *et al*.^33^. After a 16 h incubation at 22 °C in the dark, GFP signal was detected by a Zeiss LSM 700 fluorescence confocal microscopy.

### The construction of transgenic Arabidopsis thaliana

The full-length coding sequence was ligated into *pSTART* driven by the CaMV 35S promoter, and the construct was introduced into *Arabidopsis thaliana* Col-0 ecotype to overexpress using the floral dip method^34^. The stable integration of transgene in *Arabidopsis thaliana* genome was confirmed by PCR with genomic DNA as template. The transcription of the transgene was detected by real-time PCR with cDNA as template.

### Salt tolerance assay of transgenic Arabidopsis thaliana

Surface-sterilized *Arabidopsis thaliana* seeds were plated on half strength Murashige and Skoog (MS) agar medium, kept in the dark at 4 °C for two days to break dormancy, and subsequently moved to a 16 h photoperiod (2000 Lux) at 22 °C for two days. They were then re-plated on half strength MS agar medium supplemented with 0, 50 or 100 mM NaCl, and held under a 16 h photoperiod (2000 Lux) at 22 °C for twelve days.

## Results

### SR3 had superior photosynthesis activity and higher JA/ABA contents

**Figure 1:**
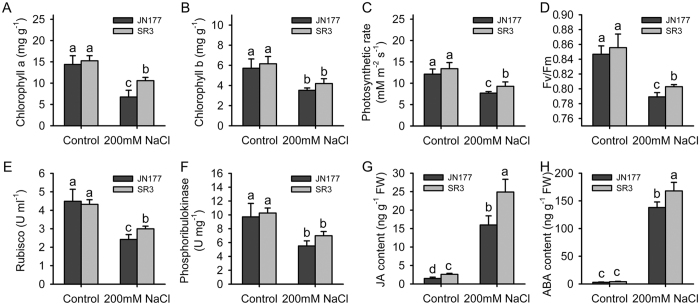
The chloroplast-associated physiological indices in leaves of wheat seedlings. (**A**) The content of chlorophyll a. (**B**) The content of chlorophyll b. (**C**) Photosynthetic rate. (**D**) The maximal quantum efficiency. (**E**) The activity of ribulose bisphosphate carboxylase/oxygenase. (**F**) The activity of phosphoribulokinase. (**G**) The JA content. (**H**) The ABA content. Data presented as mean ± standard deviation. Columns marked without different letters indicate no difference in means using the one-way ANOVA LSD analysis (*P* < 0.05).

Under the control condition, the contents of both chlorophyll a and b in SR3 were similar to those in JN177 (Fig. 1A,B). After exposure to salt stress, the contents of two types of chlorophyll were reduced, but the contents of chlorophyll in SR3 were higher than those in JN177. The photosynthetic rate of SR3 was not different from that of JN177 under the control condition, but the rate was reduced under salt stress with a stronger reduction extent in JN177 than in SR3 (Fig. 1C). SR3 had similar maximal quantum efficiency (*Fv/Fm*) compared to JN177 under the control condition (Fig. 1D). The efficiency was inhibited by salt stress, and the restriction was more obvious in JN177 than in SR3. These results indicated that photosynthesis activity was higher in SR3 than in JN177 under salt stress. Ribulose bisphosphate carboxylase/oxygenase (Rubisco) and phosphoribulokinase are two key enzymes of Calvin cycle. The activities of these two enzymes were both similar between two cultivars under the control condition; after exposure to salt stress, the activities were reduced with a less reduction extent in SR3 than in JN177 (Fig. 1E,F). We further measured the content of JA and ABA, because they are two crucial stress associated phytohormones and synthesized in chloroplast. In comparison with JN177, SR3 had higher JA contents under both the control condition and 200 mM NaCl treatment (Fig. 1G). Under the control condition, SR3 and JN177 had comparable ABA content, but after treated with 200 mM NaCl, SR3 had higher content than JN177 (Fig. 1H).

### Isolated chloroplast was qualified

**Figure 2:**
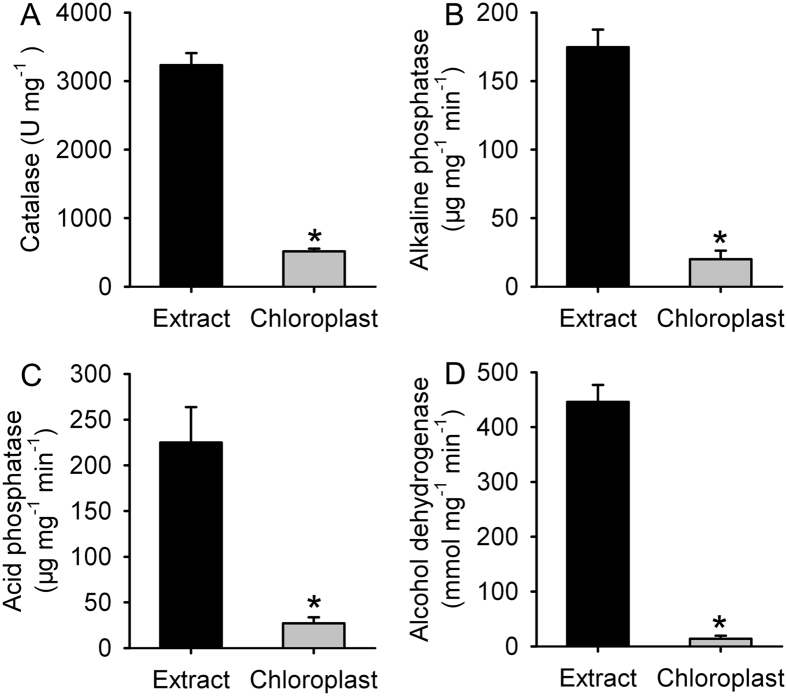
The activities of organelle marker enzymes in leaf extract and isolated chloroplast of SR3. Extract: the total proteins extracted from leaves; Chloroplast: the proteins extracted from isolated chloroplast. (**A**) The activity of catalase localizing in peroxisome. (**B**) The activity of alkaline phosphatase localizing in cell membrane. (**C**) The activity of acid phosphatase localizing in lysosome. (**D**) The activity of alcohol dehydrogenase localizing in cytosol. Data presented as mean ± standard deviation. *means the difference between extract and chloroplast was significant using the *t*-test (*P* < 0.05).

High quality of chloroplast is prerequisite for proteomic analysis. To detect the purity of chloroplast, we compared the activities of several organelle marker enzymes using SR3 samples. In comparison with the leaf cell extract, the activity of peroxisome specific enzyme catalase was lower by 84% in isolated chloroplasts (Fig. 2A). The activity of alkaline phosphatase, localizing in cell membrane, was lower by nearly eight fold in isolated chloroplasts than in the leaf cell extract (Fig. 2B). The activity of acid phosphatase, the marker enzyme of lysosome, was reduced by 88% in isolated chloroplast when compared to the leaf cell extract (Fig. 2C). Isolated chloroplast had trace activity of alcohol dehydrogenase, functioning in cytosol, lower by ~97% than the leaf cell extract (Fig. 2D). These data indicated that isolated chloroplasts were qualified for proteomic analysis.

### Proteins with differential abundance were identified

**Figure 3:**
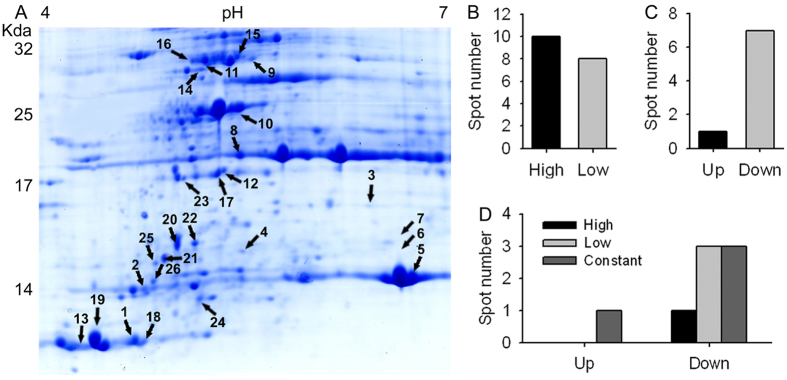
The differential expressed proteins identified by two-dimensional electrophoresis. (**A**) The reference two-dimensional electrophoresis map. The spots labeled with numbers have differential abundance among samples. (**B**) The number of cultivar-based differential expressed proteins that are not responsive to salt between SR3 and JN177. (**C**) The number of stress-responsive differential expressed proteins whose abundance was altered after exposure to NaCl treatment. (**D**) The number of stress-responsive differential expressed proteins that have different abundance between SR3 and JN177. High and low: the spots with high and low abundance in SR3 in comparison to JN177. Up and down: spots had high and low abundance after treated with NaCl.

**Figure 4:**
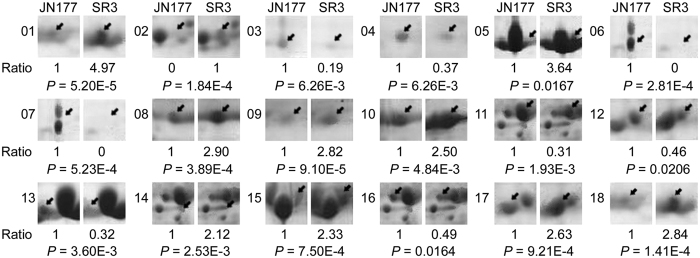
The spots of cultivar-based differential expressed proteins. The spots indicated by arrows are differential expressed proteins. Numbers at the left side of spot panels are the spots labeled in Fig. 3A and Supplementary Figure 1. Ratios below the spot panels are the relative gray values of spots in SR3 in comparison with JN177. *P* values show the significant difference in spot abundance using the *t-*test.

**Figure 5:**
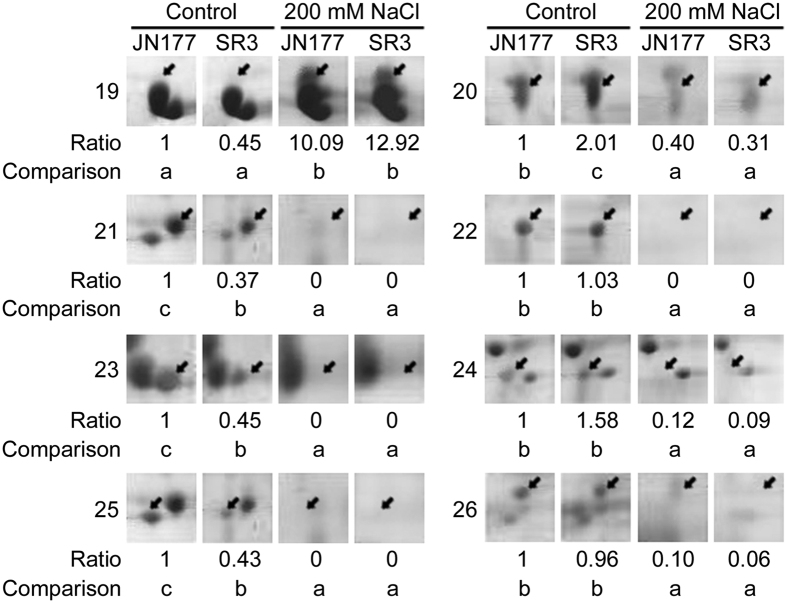
The spots of stress-responsive differential expressed proteins. The spots indicated by arrows are differential expressed proteins. Numbers at the left side of spot panels are the spots labeled in Fig. 3A and Supplementary Figure S1. Ratios below the spot panels are the relative grey values of spots with the grey values of JN177 under the control condition as the reference. The spots between two samples marked without different letters indicate no difference in means using the one-way ANOVA LSD analysis (*P* < 0.05).

The 2-DE map consisted of at least 546 reproducible protein spots, of which 26 were identified as differentially expressed proteins (DEPs) that were defined as the ratios of grey values greater than 2, on the basis of three replicated separations (Fig. 3A; Supplementary Figure S1). Among 26 DEPs, 18 were cultivar-based DEPs, who had different abundance between SR3 and JN177 but were not responsive to salt stress (spots with *p*I > 6.5 and molecular weight > 30 Kda were not selected to compare difference significance under salt stress because of 2-DE quality), of which ten (spot 1, 2, 5, 8, 9, 10, 14, 15, 17, 18) had higher abundance and eight (spot 3, 4, 6, 7, 11, 12, 13, 16) lower in SR3 (Figs 3B and 4). The other eight were stress responsive DEPs, who had either different or similar abundance between two cultivars and were responsive to salt stress (Fig. 3C). Of the salt responsive DEPs, one DEP (spot 19) was up-regulated with higher abundance after the treatment, whereas the other seven DEPs (spots 20, 21, 22, 23, 24, 26, 16) were down-regulated with lower abundance after NaCl treatment (Figs 3C and 5). The up-regulated DEP (spot 19) had trace abundance under the control condition and induced by more than ten fold by NaCl treatment, but the abundance under both non-stressful and stressful conditions was comparable between SR3 and JN177 (Fig. 5). Among seven down-regulated DEPs, one (spot 20) had higher abundance, three (spots 21, 23, 25) had lower and the other three had similar (spots 22, 24, 26) in SR3 in comparison with JN177 under the control condition, while three had similar abundance between two cultivars after exposure to NaCl (Figs 3D and 5).

Of 26 DEPs, 21 were identified, and the other five were not (Table 1; Supplementary Table S1). These identified proteins were classified into four categories. The first category was photosynthesis, including three phosphoribulokinases (spots 11, 15 and 16), three Rubisco (spots 5, 6 and 7), and oxygen-evolving enhancer protein 2 (spot 8). The second category was the stability of photosynthesis system, including a chlorophyll a/b-binding apoprotein CP24 precursor (spot 14), two 2-Cys peroxiredoxin BAS1 (spots 17 and 23), a photosystem II stability/assembly factor HCF136 (spot 9), one cytochrome P450 (spot 20). The third category was associated with protein synthesis and transporting, including chloroplast ribosome components 50S ribosomal protein L16 (spots 4, 10, 18, 24) and 30S ribosomal protein S3 (spot 26), maturase K (spot 21), signal recognition particle 54 kDa protein 1 (spot 25). The fourth category had two phospholipase A1-IIdelta (spots 2 and 3), who modulate the process of linolenic acid metabolism.

### The expression level of genes encoding some identified proteins were response to salt stress

**Figure 6:**
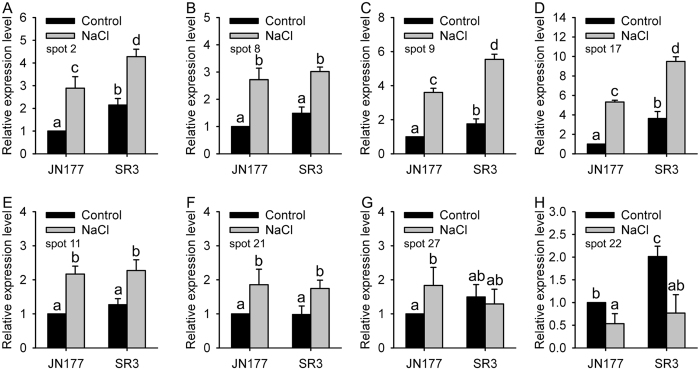
The transcriptional profiles of eight randomly selected differential expressed proteins. Relative expression level is defined as the ratio of the transcriptional level to the transcriptional level of JN177 under the control condition. Data presented as mean ± standard deviation. Columns marked without different lowercase letter indicate no difference in means using the one-way ANOVA LSD analysis (*P* < 0.05).

To understand the relationship between the abundance of a protein and the level of its gene’s transcript, we measured the expression profiles of genes encoding eight randomly selected proteins. Among cultivar-based DEPs, spots 2, 9, 11 and 17 had higher abundance in SR3 (Fig. 4), and the transcriptional levels of those related genes also were more in SR3 than in JN177 under the control condition; moreover, the expression of these genes were induced by salt stress, and was still higher in SR3 (Fig. 6A,C–E). Note that spots 3 and 2 were both identified as phospholipase A1-IIdelta, but spot 3 had lower abundance in SR3 compared to spot 2 (Fig. 4), as was also found for spots 11, 15 and 16, who were identified as phosphoribulokinase but showed opposite abundance patterns. In comparison with JN177, cultivar-based DEP spot 8 accumulated more strongly in SR3, but its corresponding gene had similar transcription level in two cultivars and was induced with similar fold when exposed to salt stress (Fig. 6B). Similar to protein abundance, the corresponding gene encoding salt-responsive DEP spot 19 was induced by salt stress with similar abundance between SR3 and JN177 (Fig. 6F). The consistent change between transcriptional and translational levels was also found in spot 20, whose corresponding gene had higher transcript abundance in SR3 and was restricted when treated with salt (Fig. 6H). The transcriptional pattern of corresponding gene encoding spot 25 was different between SR3 and JN177, and was induced by salt in JN177 but kept invariant in SR3 (Fig. 6G). These data indicates that the proteomic and transcriptomic profiles do not thoroughly coincide with each other.

### Identified proteins localized in chloroplast

**Figure 7:**
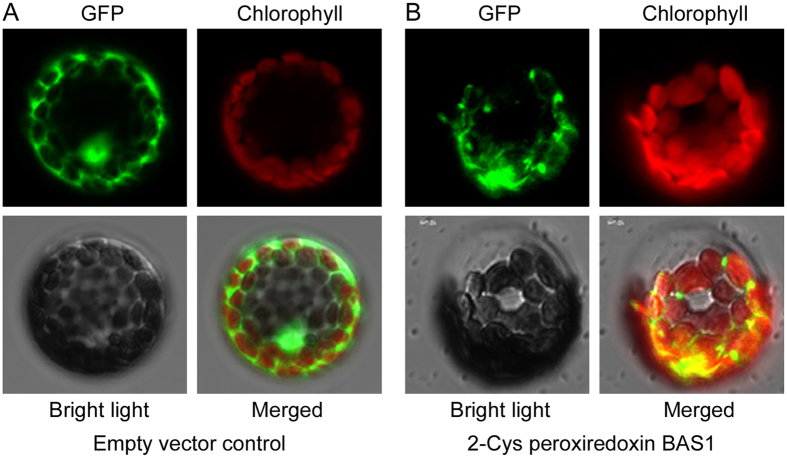
The subcellular localization assay of wheat 2-Cys peroxiredoxin BAS1 (spot 17). (**A**) Protoplast transformed with an empty vector control expressing GFP alone. (**B**) Protoplast expressing the fused protein of wheat 2-Cys peroxiredoxin BAS1 and GFP. GFP: GFP fluorescence signal; Chlorophyll: chlorophyll autofluorescence signal; Bright light: the field of bright light; Merged: the emergence of GFP fluorescence signal, chlorophyll autofluorescence signal and bright light field.

These identified proteins were predicted to localize in the chloroplast. To confirm the predication, we selected four identified proteins including oxygen-evolving enhancer protein 2 (spot 8), photosystem II stability/assembly factor HCF136 (spot 9), phosphoribulokinase (spot 11) and 2-Cys peroxiredoxin BAS1 (spot 17), respectively, and made transiently expression of their translational chimeric genes with GFP in *Arabidopsis thaliana* protoplasts via the protoplast transforming. The GFP green fluorescence was dispersed in the cytoplasm and nucleus of protoplasts expressing GFP alone (Fig. 7A). Oppositely, the GFP green fluorescence was overlapped with the chlorophyll auto-fluorescence in protoplasts transformed with 2-Cys peroxiredoxin BAS1 (Fig. 7B) and the other three genes (Supplementary Figure S2), indicating that these proteins localized in chloroplast.

### An identified DEP enhanced salt tolerance

**Figure 8:**
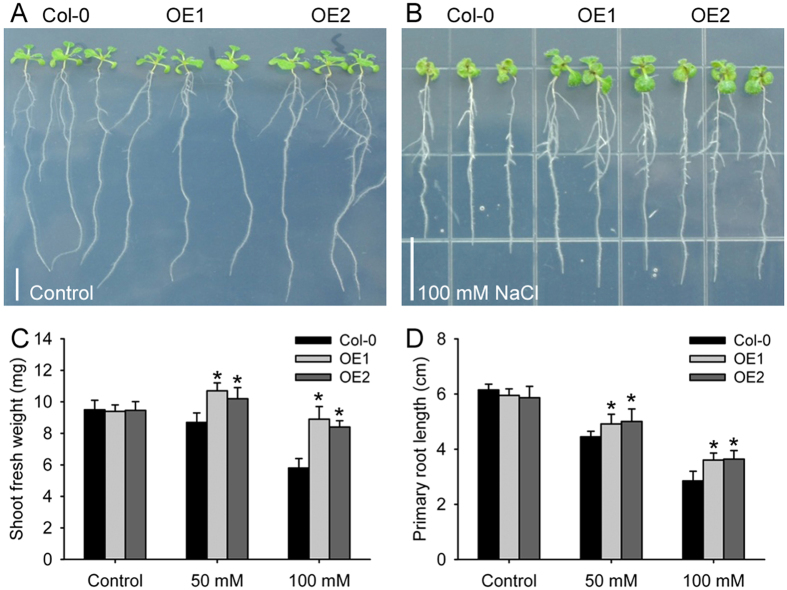
Overexpression of a wheat 2-Cys peroxiredoxin BAS1 (spot 17) enhances salt tolerance in *Arabidopsis thaliana*. (**A,B**) Two-day-old *Arabidopsis thaliana* seedlings were subject with none or NaCl treatment for twelve days. (**C,D**) The statistical results of shoot fresh weight (**C**) and root length (**D**) in panels (**A,B**). Data presented as mean ± standard deviation, and in each column set, *indicates significant difference from the control condition using the *t*-test (*P* < 0.05).

To know whether the DEPs identified from proteomic comparison analysis have potential to be used for application, we made the overexression construction of the gene encoding 2-Cys peroxiredoxin BAS1 and then transformed it into *Arabidopsis thaliana* Col-0. Two independent transgenic overexpression (OE) lines were screened and then selected for further analysis. Under the control condition, there were no difference between Col-0 and the OE lines (Fig. 8A). After exposure to different concentrations of NaCl, the growth of the seedling of both Col-0 and the OE lines were inhibited, but the OE lines exhibited stronger growth ability than Col-0 (Fig. 8B). In comparison with Col-0, the OE lines had longer roots and heavier shoot under both 50 and 100 mM NaCl treatments (Fig. 8C,D). Moreover, salt treatment delayed seed germination, but the delay was alleviated in the OE lines (data not shown). The results showed that overexpression of 2-Cys peroxiredoxin BAS1 enhanced salt tolerance of transgenic plants and suggested that the gene encoding 2-Cys peroxiredoxin BAS1 was a candidate for molecular breeding.

## Discussion

Salinity is one of the most common abiotic stresses affecting crop productivity, so to improve understanding the plant response to this environmental challenge is a major research priority. As the site of photosynthesis and production of some phytohomones and other metabolites, chloroplast is closely associated with plant growth and abiotic stress response. We previously compared the proteomes of leaves and roots, and concluded the basis of SR3’s salt tolerance, one of which is the superior assimilation recovering capacity under stressful conditions^20^. Here, we compared the chloroplast proteomes of SR3 and JN177, and uncovered the cause of the superior assimilation recovering capacity of SR3.

Firstly, the photosynthesis system has higher efficiency in SR3 compared to that in JN177. The efficiency of photosynthesis in chloroplast is mainly determined by the capacity of Calvin cycle, which play crucial roles in the machinery of abiotic stress response^35^. Phosphoribulokinase and ribulose bisphosphate carboxylase (Rubisco) are two key enzymes in Calvin cycle; the former catalyzes the conversion of ribulose 5-phosphate into ribulose 1, 5-diphosphate in the presence of ATP, and the latter catalyzes the ribulose 1, 5-diphosphate and CO_2_ into glycerate-3-phosphate. Among three spots (5, 6, 7) of ribulose bisphosphate carboxylase, two with larger molecular weight and lower *p*I value were detected in JN177, and the other one with smaller molecular weight and higher *p*I value had more abundance in SR3 (Figs 3A and 4; Supplementary Figure S1). Similarly, three spots (11, 15, 16) were identified as phosphoribulokinase; they have similar molecular weight, while the spot with the highest *p*I value had more abundance in SR3 (Fig. 4). This suggests that these proteins may have different post-translational modification (PTM) in two cultivars. Both Rubisco and phosphoribulokinase can be diversely modified, and then were modulated to the degradation, proteolysis and activity of the enzymes^36^,^37^,^38^,^39^. More importantly, the change in the abundance and PTM of these two proteins have been found in plants exposed to salt^40^. Thus, the putative PTM difference of these two key enzymes may offer the enhancement of photosynthesis efficiency of SR3 (Fig. 1C,D). Note that wheat is an allohexaploidy; its genomes come from three ancestors, so most of genes have three allelic loci. The expression of allelic genes is controlled by both genetic and epigenetic regulatory machinery during the period of wheat evolution^41^. Our previous study indicated that high frequencies of genetic and epigenetic variation is occurred in the genome of SR3 in comparison with JN177^14^,^15^, which led to the alteration of expression of some genes that are closely associated with salt response^16^,^42^. Thus, it could not be excluded that the altered abundance of different spots of a certain enzyme (encoded by nuclear genes) is attributed to the altered expression of its allelic genes that is caused by genetic and epigenetic variation. In line with higher activities of these two enzymes in SR3 than in JN177 (*P* value was 0.057 for phosphoribulokinase activity between SR3 and JN177 under salt stress) (Fig. 1E,F), the data indicate that the difference in their abundance enhances photosynthesis efficiency of SR3 under salt treatment.

Secondly, the capacity for the stability of photosynthesis system is superior in SR3. Photosystem II (PSII) stability/assembly factor HCF136 (spot 9) had higher abundance in SR3 (Fig. 4). HCF136 is a hydrophilic protein localized in the lumen of stroma thylakoids. Its mutational inactivation in *Arabidopsis thaliana* results in a PSII-less phenotype, and biogenesis of the reaction center of PSII is blocked^43^. Thus, the accumulation of HCF136 in SR3 contributes the superior photosynthesis ability of SR3. In line with the finding that HCF136 is down-regulated by Mn and powdery mildew^44^, it could be suggested the complicated function in the response to environmental stimuli. The abundance of the chlorophyll a/b-binding apoprotein CP24 precursor (spot 14) in SR3 is > two fold than in JN177 (Fig. 4). This protein is a member of a chlorophyll binding complex which protects chlorophyll in cells, and plays an important role in photosynthesis^45^. It enhances drought tolerance via modulating H_2_O_2_ level in guard cells to promote stomatal close^46^. In combination with higher chlorophyll content in SR3 (Fig. 1A,B), the conclusion is that SR3 has stronger capacity of chlorophyll protection to get higher photosynthesis rate under salt treatment (Fig. 1C,D).

ROS is often produced following electron transfer process during photosynthesis^4^,^5^, and its over-accumulation should be removed to avoid toxicity and to maintain the redox status, because the suitable redox status of chloroplasts is crucial in biological stress response and helps the plant to cope with environmental changes^47^. 2-Cys peroxiredoxins serve as not only anti-oxidants but also chaperones and regulatory proteins to participate in signalling transduction of redox signals and other processes^48^. A 2-Cys peroxiredoxin enhanced tolerance to methyl viologen and heat stresses in transgenic tall fescue^49^. Here, a 2-Cys peroxiredoxin BAS1 (spot 17) has higher abundance in SR3 (Fig. 4), and its overexpression enhances salt tolerance in *Arabidopsis thaliana* (Fig. 8), indicating that higher abundance of this protein may partially contribute to the slat tolerance of SR3. Moreover, 2-Cys peroxiredoxin can directly activate ROS production enzyme NADPH oxidase and ROS scavenging enzymes such as glutathione peroxidases (GPX) and NADPH-dependent thioredoxin reductase (NTRC)^50^,^51^. We previous found that SR3 has higher ROS level and the activities of NADPH oxidase and some ROS scavenging enzymes^17^. In line with our previous study^20^, these results provides a strong evidence for the crucial role of superior redox homeostasis maintenance capacity in salt tolerance of SR3. Note that spot 23 identified as 2-Cys peroxiredoxin BAS1 has lower abundance in SR3 and is inhibited after exposure to salt. In *Arabidopsis thaliana*, the diverse roles of 2-Cys peroxiredoxin members and the effect of the post-translational modification on their roles have been found (See review in^52^,^53^). Here, spots 17 and 23 have similar molecular weight but different *p*I values, so whether they are two homologues of 2-Cys peroxiredoxin BAS1 or two modification types of the same 2-Cys peroxiredoxin BAS1 as well as the difference and similarity of their contribution to salt response needs to be further investigated.

Thirdly, the synthesis of stress-associated phytohormones is enhanced in SR3. Besides photosynthesis, chloroplast is the site for synthesis of stress associated phytohormones such as JA and ABA. Phospholipases catalyze the metabolism of phospholipids, which are important signalling molecules participating in abiotic stress response pathway. In plants, a chloroplast-localized phospholipase A, DAD1, is expected to be responsible for release of linolenic acid from membrane lipids, the first step of JA synthesis, and its transcript was maximally induced 1 h after wounding^54^. Phospholipase A has also been implicated in wound-, and systemin-induced JA formation in tomato^55^. Besides, other chloroplast-localized lipases related to DAD1 have been identified, and one or more of these might be involved in wound-induced JA biosynthesis^54^. Here, two DEPs, spot 2 and 3, with similar *p*I values but different molecular weight are Phospholipase A1-IIdelta (Fig. 4; Table 1), and they have higher and lower abundance respectively in SR3 (Fig. 4). In line with higher JA content in SR3 than in JN177 (Fig. 1G), it could be concluded that SR3’s salt tolerance is associated with stronger JA synthesis. Consistently, our previous transcriptomic analysis found the genes of several enzymes catalyzing different steps of α-linolenic acid metabolism pathway had differential expression patterns between SR3 and JN177^19^. Of them, the overexpression of wheat allene oxide cyclase gene *TaAOC1*, catalyzing JA synthesis (JA-branch), significantly enhanced salt tolerance in wheat and *Arabidopsis thaliana*^56^; in parallel, the overexpression of wheat 12-oxophytodienoic acid reductase gene *TaOPR1*, catalyzing OPR-branch of linolenic acid methabolism pathway, also offered wheat and *Arabidopsis thaliana* salt tolerance^57^. Notably, although ABA synthesis associated proteins are not identified, SR3 has higher ABA content under salt treatment (Fig. 1H), as suggests SR3 chloroplast possesses superior ABA synthesis activity.

Lastly, the transcription and translation system shows difference between SR3 and JN177. Maturase K (Spot 19) is drastically induced under salt stress (Fig. 5). Maturase K is involved in the splicing of pri-mRNA into mature mRNA. In plants, maturase K preferentially catalyzes the binding of the intron RNA during reverse transcription and splicing^58^. The editing of mRNA prevails in plastidic genes, and performs crucial role in regulating gene function. Almost all land plant chloroplasts, with the exception of some parasitic species in the genus *Cuscuta*, contain a single maturase gene called matK in the intron of the lysine tRNA-K (UUU) gene^59^. However, a maturase K is found to be dephosphorylated after adjustment to saline conditions^60^. Here, the spot below spot 19 is also identified as maturase K, implying maturase K may be modified under stressful conditions in wheat. These findings indicate that maturase K is certainly involved in the response to salt treatment via modulating the process of mRNA splicing. The chloroplast equivalent of the signal recognition particle pathway (CpSRP) catalyzes the post-translational and co-translational protein insertion into the lipid bilayer of the developing thylakoid membrane^61^. The *chaos* mutant of *Arabidopsis thaliana*, which is unable to accumulate ELIPs during light stress for lacking cpSRP43 (a subunit of the cpSRP complex), is more sensitive to light stress than the wild-type^62^. However, signal recognition particle (SRP) 54 kDa protein 1 (spot 25) has lower abundance in SR3 and is down-regulated by salt (Fig. 5), showing the complicated performance of signal recognition particle in salt response. Furthermore, a set of cultivar-specific (spots 4, 10, 18) and salt responsive (spots 24, 26) ribosome proteins (Figs 4 and 5) indicate the close association between protein synthesis of chloroplast genes and salt response.

In summary, this proteomic analysis gives us a comprehensive insight into the association of chloroplast with the high salt tolerance of SR3, which may be a global capacity ranging from higher photosynthesis efficiency by promoting system protection and ROS clearance, increased production ability of phytohormones JA and ABA by enhancing metabolism activity, to modulating the synthesis of proteins encoding by the chloroplast genome. Besides, the enhanced salt tolerance by overexpressing the DEP 2-Cys peroxiredoxin BAS1 indicates that the proteomic analysis is an efficient strategy for mining candidate genes for crop molecular breeding.

## Additional Information

**How to cite this article**: Xu, W. *et al*. Proteomic comparison reveals the contribution of chloroplast to salt tolerance of a wheat introgression line. *Sci. Rep.* **6**, 32384; doi: 10.1038/srep32384 (2016).

## Supplementary Material

Supplementary Information

Supplementary Information

## Figures and Tables

**Table 1:** The identification of differentially expressed proteins.

Spot	Accession No.	Annotation	Species	Tmw	Emw	TpI	EpI	Score
2	PLA20_ARATH	Phospholipase A1-IIdelta	*Arabidopsis thaliana*	46.03	14.34	5.11	4.72	64
3	PLA20_ARATH	Phospholipase A1-IIdelta	*Arabidopsis thaliana*	46.03	16.01	5.11	6.24	63
4	RK16_NANDO	50S ribosomal protein L16	*Nandina domestica*	15.27	15.11	11.39	5.37	53
5	RBS_HORVU	Ribulose bisphosphate carboxylase small chain	*Hordeum vulgare*	19.41	14.36	8.98	6.54	97
6	RBS3_WHEAT	Ribulose bisphosphate carboxylase small chain clone 512 (Fragment)	*Triticum aestivum*	13.05	15.2	5.84	6.47	119
7	RBS3_WHEAT	Ribulose bisphosphate carboxylase small chain clone 512 (Fragment)	*Triticum aestivum*	13.05	15.63	5.84	6.47	76
8	PSBP_WHEAT	Oxygen-evolving enhancer protein 2	*Triticum aestivum*	27.25	19.01	8.84	5.35	61
9	gi|475519847	photosystem II stability/assembly factor HCF136	*Aegilops tauschii*	41.83	29.58	6.15	5.43	76
10	RK16_COFAR	50S ribosomal protein L16	*Coffea arabica*	15.43	25.37	11.89	5.34	65
11	KPPR_WHEAT	Phosphoribulokinase	*Triticum aestivum*	45.11	29.46	5.72	5.13	88
12	gi|168057698	predicted protein	*Physcomitrella patens*	15.83	17.31	7.85	5.25	72
14	T02253	chlorophyll a/b-binding apoprotein CP24 precursor	*Zea mays*	27.01	27.43	9.24	5.14	121
15	KPPR_WHEAT	Phosphoribulokinase	*Triticum aestivum*	45.11	29.77	5.72	5.32	70
16	KPPR_WHEAT	Phosphoribulokinase	*Triticum aestivum*	45.11	29.77	5.72	5.04	97
17	BAS1_HORVU	2-Cys peroxiredoxin BAS1	*Hordeum vulgare*	23.28	16.96	5.48	5.18	63
18	RK16_SACHY	50S ribosomal protein L16	*Saccharum hybrid*	15.51	13.22	11.56	4.67	52
19	MATK_CLEFL	Maturase K	*Clematis florida*	61.05	13.4	9.55	4.46	61
20	C82C2_ARATH	Cytochrome P450	*Arabidopsis thaliana*	58.96	15.23	8.69	4.97	63
23	BAS1_HORVU	2-Cys peroxiredoxin BAS1	*Hordeum vulgare*	23.28	16.89	5.48	5.02	60
24	RK16_CALFG	50S ribosomal protein L16	*Calycanthus floridus*	15.40	13.86	11.56	5.13	41
25	SR541_SOLLC	Signal recognition particle 54 kDa protein 1	*Solanum lycopersicum*	54.79	14.79	9.19	4.82	51
26	RR3_CITSI	30S ribosomal protein S3	*Citrus sinensis*	25.27	14.33	9.95	4.81	57

Spot: The numbers of the proteins labeled in 2-DE reference gel of Fig. 3. Accession No: The NCBInr, MSDB or SwissProt reference numbers of proteins. Tmw: The molecular mass of the predicted protein. Emw: The molecular mass of the protein as estimated by its gel migration. TpI: The isoelectric point of the predicted protein: EpI: the isoelectric point of the protein as estimated by its gel migration. Score: The score of credible identification in Mascot using MS data.
