# Supplementary material for: Proteomic comparison reveals the contribution of chloroplast to salt tolerance of a wheat introgression line
Source: Sci Rep. 2016 Aug 26;6:32384. doi: 10.1038/srep32384 (PMC4999883; doi:10.1038/srep32384)
Supplement: Supplementary Information [file srep32384-s1.pdf]

Supplementary Files for

Proteomic comparison reveals the contribution of chloroplast to salt tolerance of a wheat introgression line

Wenjing Xu, Hongjun Lv, Mingming Zhao, Yongchao Li, Yueying Qi, Zhenying Peng, Guangmin Xia, Mengcheng Wang

Supplementary Table S1. The MASCOT searching results of differentially expressed proteins. (Provided as a separate supplementary dataset)

Supplementary Figure S1. The two-dimensional electrophoresis maps of SR3 and JN177 under the control condition and salt stress.

Supplementary Figure S2. The subcellular localization assay of three selected DEPs.

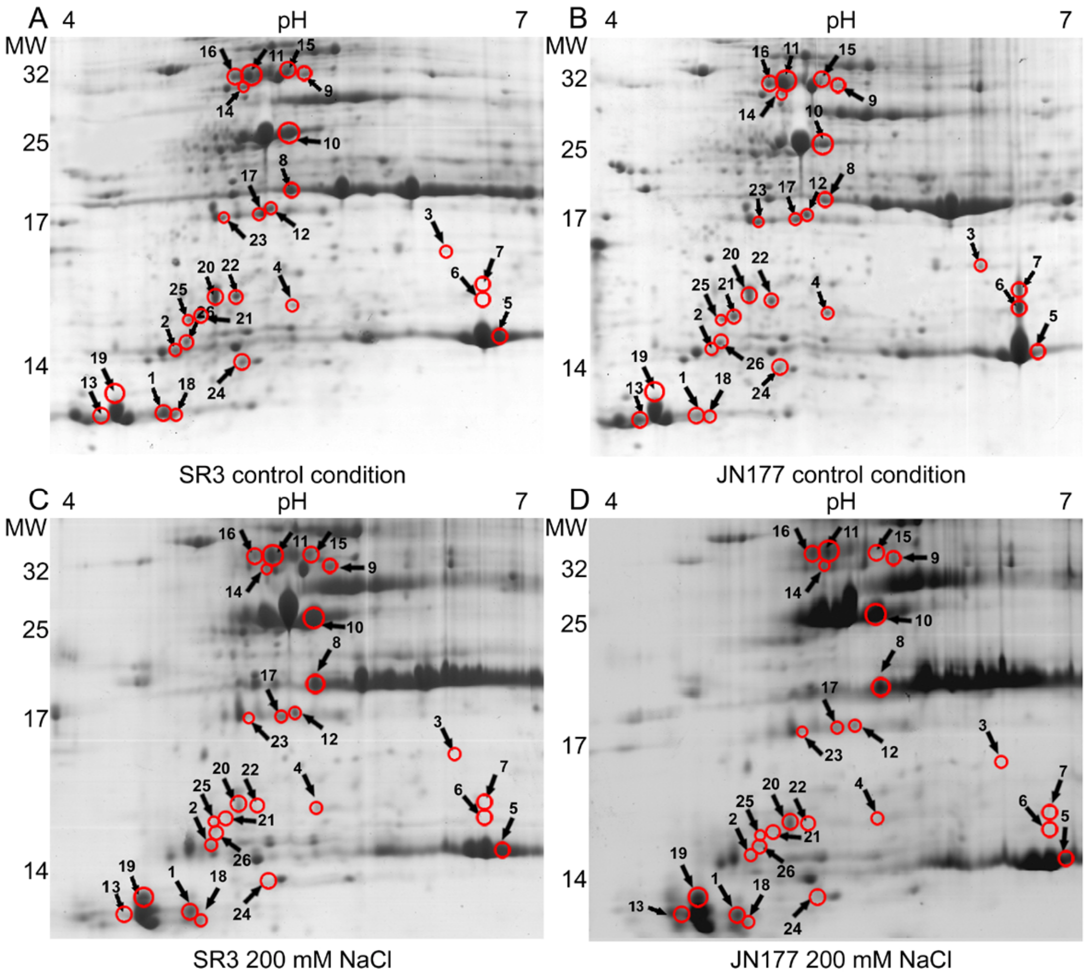

Supplementary Figure S1. The two-dimensional electrophoresis maps of SR3 and JN177 under the control condition and salt stress. A: SR3 under the control condition. A: JN177 under the control condition. C: SR3 under 200 mM NaCl treatment. D: JN177 under 200 mM NaCl treatment. The spots labeled with numbers have differential abundance among samples. Red circle: the corresponding regions of DEPs in four maps.

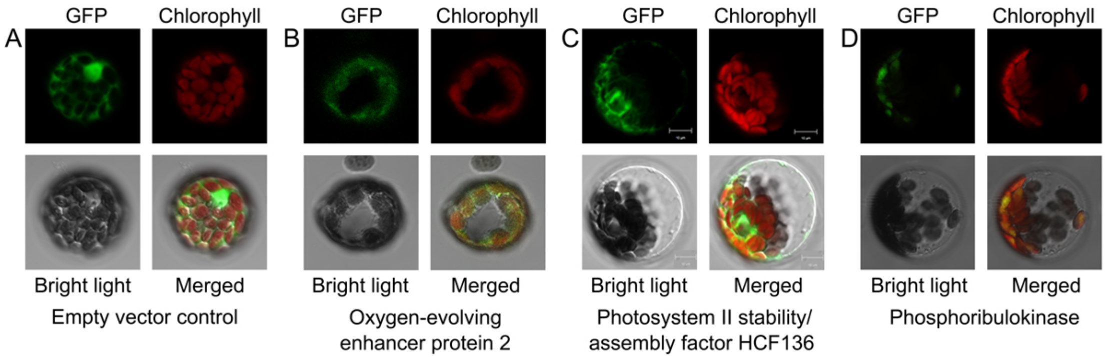

Supplementary Figure S2. The subcellular localization assay of three selected DEPs. A: Protoplast transformed with an empty vector control expressing GFP alone. B: Protoplast expressing the fused protein of oxygen-evolving enhancer protein 2 (spot 8) and GFP. C: Protoplast expressing the fused protein of photosystem II stability/assembly factor HCF136 (spot 9) and GFP. D: Protoplast expressing the fused protein of phosphoribulokinase (spot 11) and GFP. GFP: GFP fluorescence signal; Chlorophyll: chlorophyll autofluorescence signal; Bright light: the field of bright light; Merged: the emergence of GFP fluorescence signal, chlorophyll autofluorescence signal and bright light field.
